# Monitoring of cherry flowering phenology with Google Trends

**DOI:** 10.1371/journal.pone.0271648

**Published:** 2022-07-21

**Authors:** Nagai Shin, Ayumi Kotani, Shunsuke Tei, Narumasa Tsutsumida

**Affiliations:** 1 Research Institute for Global Change, Japan Agency for Marine-Earth Science and Technology (JAMSTEC), Yokohama, Japan; 2 Graduate School of Bioagricultural Sciences, Nagoya University, Nagoya, Japan; 3 Department of Forest Management, Forestry and Forest Products Research Institute, Tsukuba, Japan; 4 Graduate School of Science and Engineering, Saitama University, Saitama, Japan; Chinese Academy of Sciences, CHINA

## Abstract

Google Trends (GT) is an online tool designed for searching for changes over time. We assessed its use for evaluating changes in the timing of cherry flowering phenology, which is of intense interest to Japanese people. We examined the relationship between time-series of relative search volume (RSV: relative change in search requests over time obtained from the GT access engine) and cherry flowering information published on websites (as ground truth) in relation to three famous ancient cherry trees. The time-series of RSV showed an annual bell-shaped seasonal variability, and the dates of the maximum RSV tended to correspond to the dates of full bloom. Our results suggest that GT allows monitoring of multiple famous cherry flowering sites where we cannot obtain long-term flowering data to evaluate the spatiotemporal variability of cherry flowering phenology.

## Introduction

Flowering by cherry trees is an important cultural harbinger of spring in Japan. Its appeal dates from imperial banquets in the 9th century AD [1], being fully rooted in Japanese traditions, culture, literature, and arts. Japanese people enjoy cherry blossoms and related events in spring, and cherry flowering has great economic value for tourism [2]. Japanese people mark the year through various kinds of plant and animal phenology [3] and are especially interested in cherry flowering dates above all.

In Japan, leaf flush of many deciduous plants occurs after cherry flowering. This timing makes cherry flowering suitable as a proxy of spring phenology. In fact, before the beginning of scientific meteorological observations, Japanese people referred to cherry flowering to plan their planting seasons. Climate change has advanced the dates of cherry flowering throughout Japan during the past 70 years [4]. Statistical flowering phenology models predict a future with late flowering, incomplete bloom, and failure of flowering in areas where the annual mean temperature is high owing to a lack of the chilling required for dormancy release [5]. Variability of plant phenology in spring strongly affects ecosystem functions (photosynthesis and evapotranspiration), nature’s contributions to people, and biodiversity [6–8]. Therefore, although accurate evaluation of plant phenology in spring is challenging, it is an important task.

The Japan Meteorological Agency (JMA) has recorded the start dates of flowering and full bloom of cherry since 1953 at weather stations throughout Japan [4]. However, the number of observation points has decreased in recent years (58 points as of 2021). In addition, there was no detailed flowering phenology data that changed daily (e.g., 50% flowering, start of scattering, and green leaves). In contrast, information on cherry flowering at many famous sites is published on websites. Meteorological organizations provide cherry flowering information at many sites throughout Japan, and local governments and tourist associations provide information at specific sites. Many websites provide updated daily cherry flowering information by text message. Some use micro-blogs, phenology images, and live cameras. To evaluate the spatiotemporal characteristics of leaf coloring along altitudinal and elevational gradients and the relationship between flowering periods and festivals under climate change, we reported the utility of flowering and leaf coloring information published on such websites [2, 9]. However, such information is overwritten daily, so long-term historical data are unavailable.

Google Trends (GT) is an online tool for searching changes over time [10]. It allows researchers to evaluate changes as time-series of relative search volume (RSV) in a given period and region, offering a unique index to represent the level of people’s interest in a topic [11]. The utility of GT has been shown in many studies, such as in the prediction of infection by COVID-19 [12], societal effects due to infection by COVID-19 [13–16], allergic rhinitis induced by Japanese cedar pollen [17], research and public interest in melanoma [18], global and country-specific interest in obesity, smoking, and alcoholism [19], societal concerns about pesticides [20], and Japanese citizens’ interest in insects [21]. However, there are few previous studies of plant phenology using this tool [22]. Despite lacking validation of ripening by using detailed *in situ* data, Kotani et al. [22] reported the utility of GT for the spatiotemporal variability of people’s interest in berries in Finland, Russia, and Canada. This fact suggests that time-series of RSV could be used as a proxy for cherry flowering phenology and its year-to-year variability.

To validate this hypothesis, we examined the relationship between time-series of RSV and cherry flowering information published on websites (as ground truth) in relation to three famous cherry trees in Japan, where we could obtain updated daily flowering phenology information. Our aim was to examine the utility of monitoring of cherry flowering phenology with GT. We expect that analysis of time-series of RSV will allow us to monitor multiple sites where we cannot obtain long-term detailed cherry flowering phenology observations.

## Materials and methods

### Study sites

**Fig 1:**
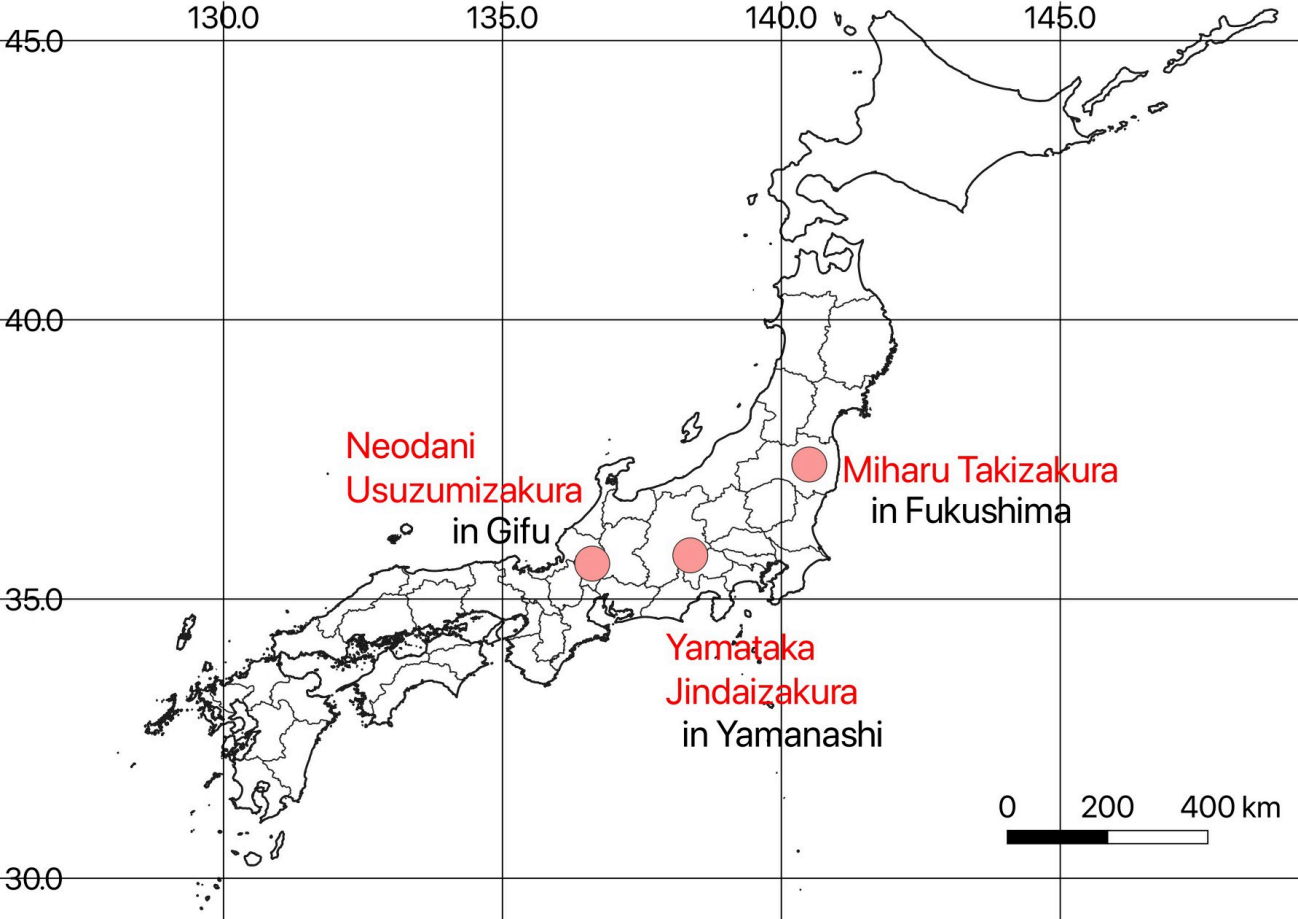
Locations of three famous cherry trees in Japan (data from Ministry of Land, Infrastructure, Transport and Tourism of Japan [27]). Latitude and longitude values were examined by using “Google Maps”.

Our targets were three famous ancient cherry trees (*Cerasus speciosa* (Koidz.) H. Ohba): Miharu Takizakura (>1000 years old, 13.5 m tall) in Fukushima (37°24′27.6″N, 140°30′00.3″E), Yamataka Jindaizakura (~2000 years old, 10.3 m tall) in Yamanashi (35°46′49.0″N, 138°22′03.9″E), and Neodani Usuzumizakura (>1500 years old, 17.3 m tall) in Gifu (35°37′56.2″N, 136°36′31.7″E) (Fig 1). These trees are designated as precious natural treasures in Japan [23–26]. We selected these trees because the search terms directly indicate the trees, not flowering sites, and we could obtain detailed flowering data published on websites.

**Fig 2:**
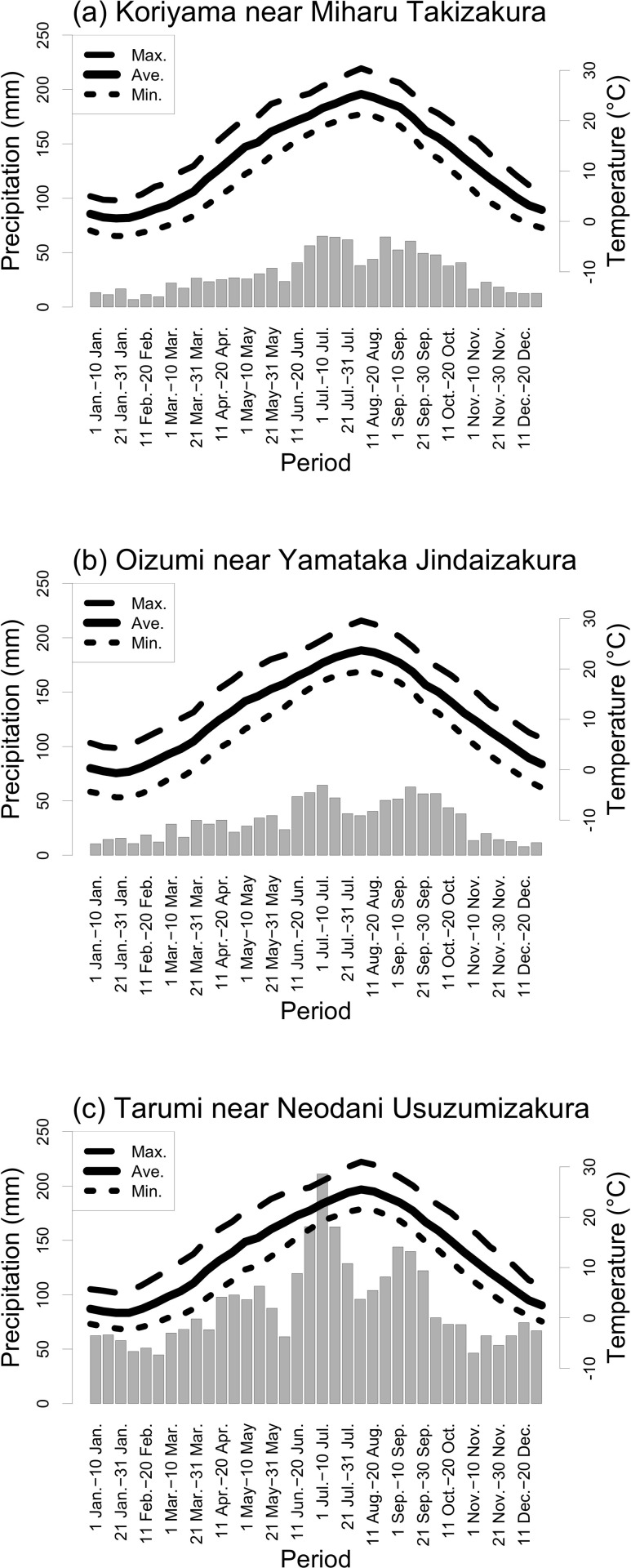
Ten-day average temperature (lines) and precipitation (boxes) based on observations from 1991 to 2020 at Koriyama AMeDAS station near Miharu Takizakura, Oizumi AMeDAS station near Yamataka Jindaizakura, and Tarumi AMeDAS station near Neodani Usuzumizakura [28].

We plotted the average observed temperature and precipitation every 10 days from 1991 to 2020 at the Koriyama Automated Meteorological Data Acquisition System (AMeDAS) station (37°22′12.7″N, 140°19′49.3″E) near Miharu Takizakura, the Oizumi AMeDAS station (35°51′41.8″N, 138°23′16.1″E) near Yamataka Jindaizakura, and the Tarumi AMeDAS station (35°38′20.8″N, 136°36′11.8″E) near Neodani Usuzumizakura (Fig 2) [28].

### Google Trends (GT) data

#### RSV

The GT search engine provides access by “Terms” and “Topics” [11]. By “Terms,” GT provides an exhaustive search for queries in a given language [18]; e.g., “三春滝桜” (“Miharu Takizakura”). By “Topics”, “predefined thematic headings group all related words, alternative spellings, and names in other languages under a single label” [29]; e.g., “Miharu Takizakura”, “三春滝桜”, and words in other languages. RSV has a temporal resolution of 1 minute, 1 hour, 1 day, 1 week, or 1 month. In a given period, RSV = 100 when the number of target queries reaches the maximum and 0 when it is less than a certain threshold [11, 22]. The target region can be specified; in Japan, the type of region with the highest spatial resolution is the prefecture. GT changed the data collection method on 1 January 2011 and 1 January 2016 [10].

We acquired RSV by “Topics” in all of Japan and by prefecture from 1 March to 30 April 2004 to 2021 (Table 1). We did not acquire RSV by “Terms” because the time-series of RSV returned includes more noise than that by “Topics” [22]. In this case, the temporal resolution is daily, and the total number when RSV = 100 is 18. However, we cannot obtain exactly the same RSV for the same request on other days because GT caches new samples every day [11]. We confirmed no effect of this issue by acquiring daily RSV on three dates (12 June, 30 June, and 5 October 2021).

**Table 1:** Query terms used to search Google Trends and flowering phenology data.

Target tree	Search term	Target period	Flowering phenology data	Target area
Miharu Takizakura	三春滝桜 /Miharu Takizakura (%2Fm%2F0 gtxdvh)*	1 Mar– 30 Apr each year	2016–2020	Japan
				Fukushima
Yamataka Jindaizakura	神代桜/Jindaizakura (%2Fg%2F121 mdt73)	1 Mar– 30 Apr each year	2004–2016	Japan
				Yamanashi
Neodani Usuzumizakura	根尾谷淡墨ザクラ/Neodani Usuzumizakura (%2Fg%2F121 gjvvc)	1 Mar– 30 Apr each year	2004–2021	Japan
				Gifu

*Characters in parentheses show the multilingual code in the GT search engine.

#### Attribute information of RSV

RSV includes information on three attributes: “interest by subregion/region,” “related queries,” and “related topics.” Each attribute has a relative score (≤100) representing search popularity rating in the results obtained in the target area and period. “Related queries” and “related topics” include two categories: “Top” and “Rising” [18]. To validate the reliability and uncertainty of time-series of RSV, we used information on all three attributes, although we used “Top related topics” and “Top related queries” (with scores of ≥30), because “Rising related topics” and “Rising related queries” are not quantitative indices.

### Flowering phenology data

As ground-truth data, we used the cherry flowering information published on websites. For Miharu Takizakura, we obtained the dates of bud swelling, start of flowering, 30% flowering, 50% flowering, full bloom, start of scattering, and green leaves (i.e., end of flowering) from 2016 to 2020 [23]. For Yamataka Jindaizakura, we obtained the dates of the start of flowering, full bloom, and green leaves from 2000 to 2016 [30]. For Neodani Usuzumizakura, we obtained the dates of the start of flowering, 20% to 30% flowering, 50% flowering, full bloom, start of scattering, and end of flowering from 1989 to 2021 [25].

We examined the relationships between (1) the seasonality of RSV and flowering stages and (2) between the year-to-year variability of the date when the time-series of RSV reaches a maximum and that of flowering phenology. We conducted all analyses in R v. 3.6.2 [31], LibreOffice v. 7.1.4.2 [32], and QGIS v. 3.10.11–A Coruña [33] software and shell scripts. To download and analyze RSV, we used the “gtrendsR” v. 1.4.8 package in R [34, 35]. We accessed GT on 5 October 2021.

## Results

### RSV

**Fig 3:**
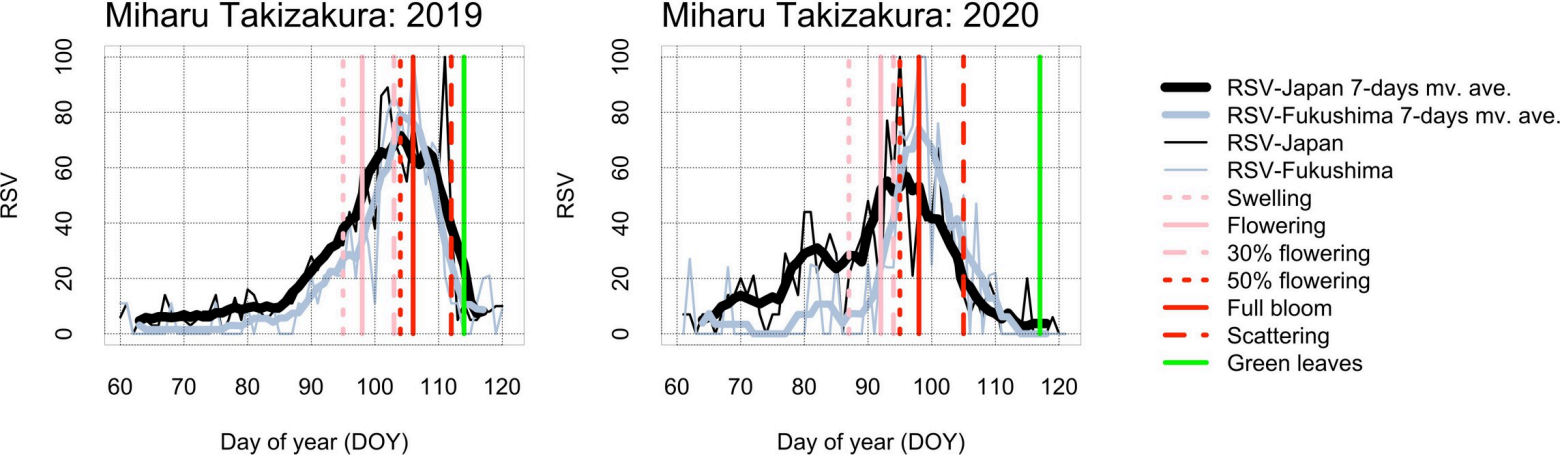
For Miharu Takizakura, the relationship between time-series of RSV searched by “Topics” in all Japan and Fukushima and flowering information published on websites from 2019 to 2020. For more result regarding the relationship from 2016 to 2018, see S1 Fig. mv. ave.: moving average.

**Fig 4:**
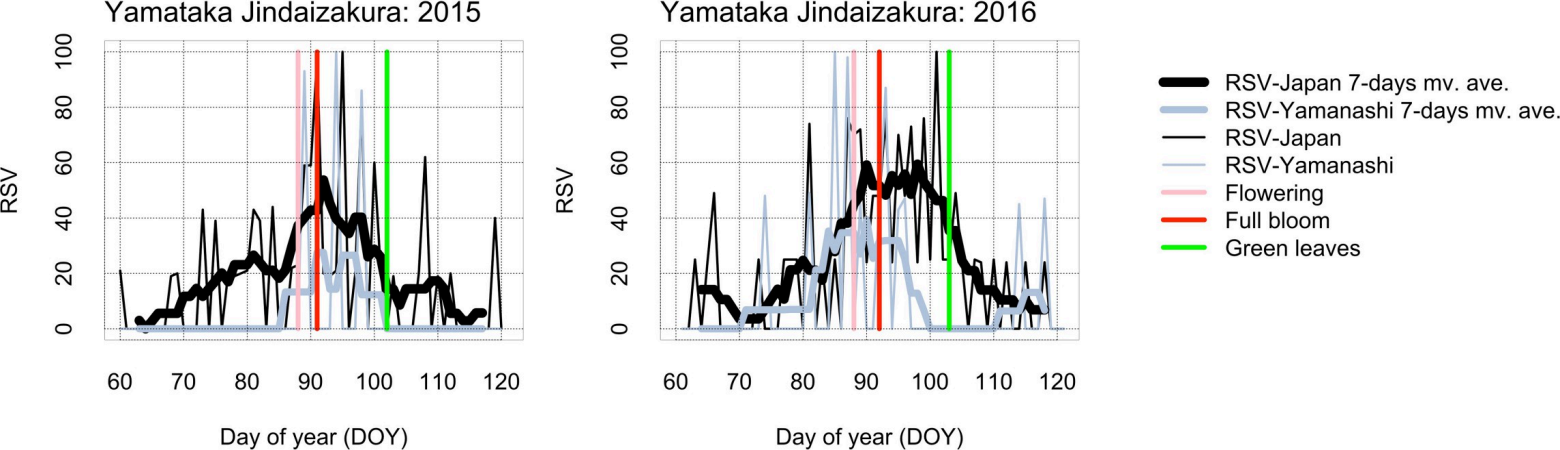
For Yamataka Jindaizakura, the relationship between time-series of RSV searched by “Topics” in all Japan and Yamanashi and flowering information published on websites from 2015 to 2016. For more result regarding the relationship from 2004 to 2014, see S2 Fig. mv. ave.: moving average.

**Fig 5:**
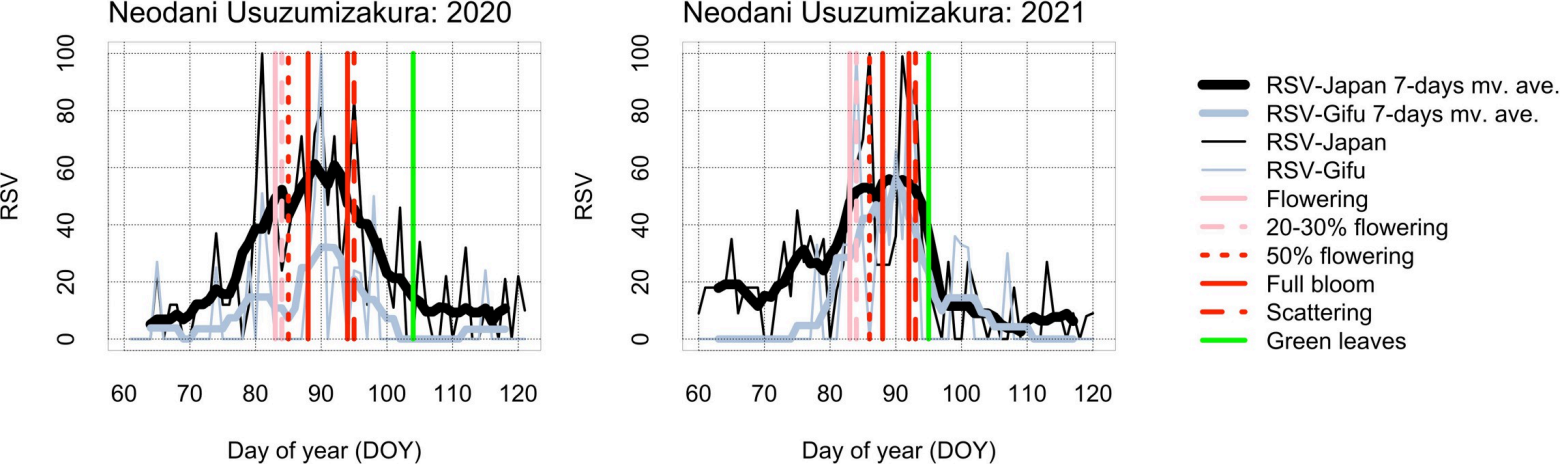
For Neodani Usuzumizakura, the relationship between time-series of RSV searched by “Topics” in all Japan and Gifu and flowering information published on websites from 2020 to 2021. For more result regarding the relationship from 2004 to 2019, see S3 Fig. mv. ave.: moving average.

**Fig 6:**
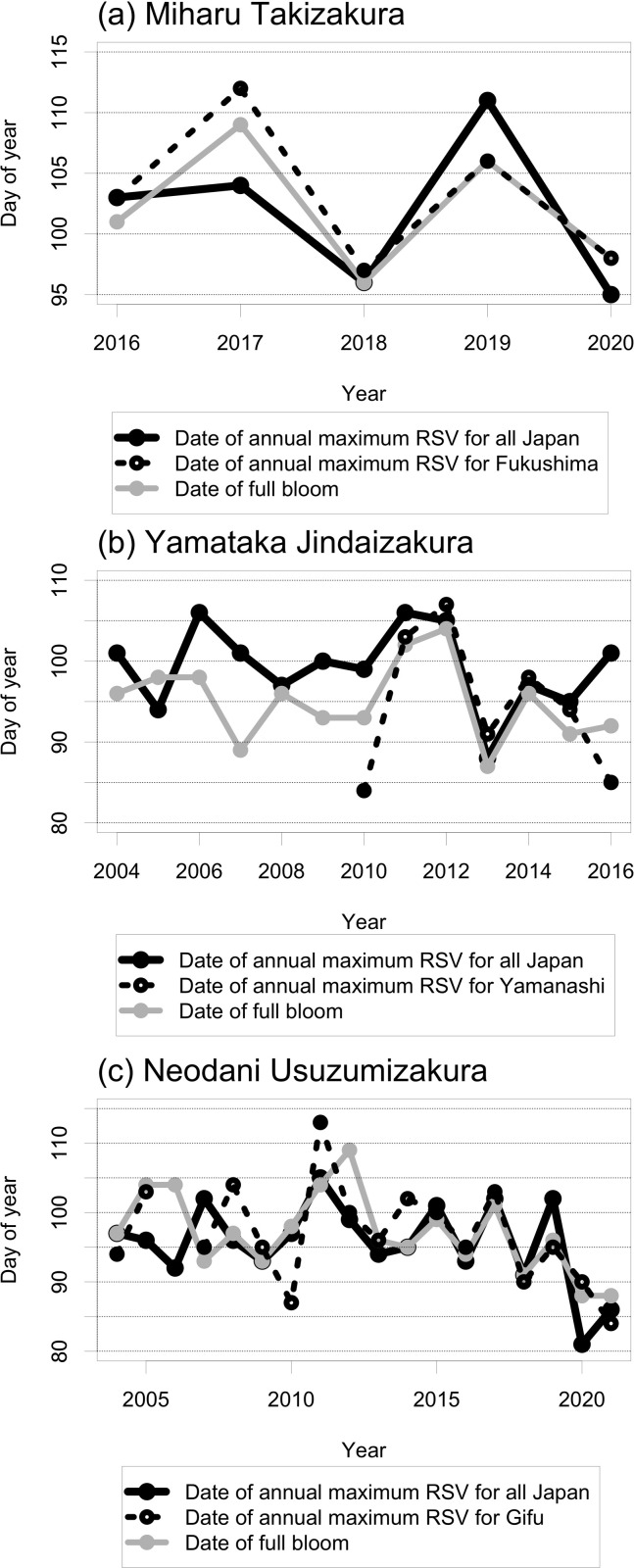
For each indicated tree, the relationship between the date of annual maximum RSV (= 100) searched by “Topics” in all Japan and by prefecture and the first date of full bloom based on the flowering information published on websites. For Miharu Takizakura in 2018 in all Japan (day of year [DOY] 96 and 97), Miharu Takizakura in 2020 in Fukushima (DOY 98 and 99), and Neodani Usuzumizakura in 2014 in Gifu (DOY 80 and 102), the dates of annual maximum RSV overlapped; we selected the date closer to the date of full bloom. See statistics in Table 2.

In the time-series of RSV searched by “Topics” from day of year (DOY) 60 (61 in leap years) to 120 (121 in leap years), seasonality of RSV was clearer in Japan than by prefecture (Figs 3–5 and S1–S3). For Miharu Takizakura, the dates of annual maximum RSV searched by “Topics” corresponded closely to the dates of flowering and full bloom (Figs 3 and S1). For Yamataka Jindaizakura, those in Japan corresponded to the dates after flowering and full bloom in many years and to the dates before flowering or after green leaves in some years (Figs 4 and S2). For Neodani Usuzumizakura, they corresponded to the dates after flowering or full bloom in many years and to the dates before flowering or after scattering in some years (Figs 5 and S3). For Miharu Takizakura and Neodani Usuzumizakura, the year-to-year variability of the date when the time-series of RSV reaches a maximum (= 100) in Japan or by prefecture was correlated with that of the first date of full bloom (Fig 6 and Table 2). The seasonality of RSV became clearer after changes in the GT algorithms on 1 January 2011 and 1 January 2016 (Figs 4 and 5 and S2 and S3; [10]). For Yamataka Jindaizakura, Spearman’s rank correlation ρ between the year-to-year variability of the date when the time-series of RSV reaches a maximum in Japan and that of the first date of full bloom from 2011 to 2016 (ρ = 0.89, *P* < 0.05) was significantly larger than that from 2004 to 2010 (ρ = −0.12, *P* = 0.80). Similarly, for Neodani Usuzumizakura, that from 2016 to 2021 (ρ = 0.97, *P* < 0.01) was significantly larger than those from 2004 to 2010 (ρ = −0.38, *P* = 0.40) and from 2004 to 2015 (ρ = 0.24, *P* = 0.46).

**Table 2:** Statistical relationship between the year-to-year variability of the date when the time-series of RSV reaches a maximum in all Japan and by prefecture and that of the first date of full bloom.

Target tree	Spearman’s rank correlation ρ			
	In all Japan		By prefecture	
Miharu Takizakura	0.80	*P* = 0.13 (5)	1.00	*P* < 0.05 (5)
Yamataka Jindaizakura	0.49	*P* = 0.09 (13)	0.68	*P* = 0.11 (7)
Neodani Usuzumizakura	0.57	*P* < 0.05 (18)	0.68	*P* < 0.01 (17)

Sample numbers are shown in parentheses.

### Attribute information of RSV

Most “Interest by region” attributes of RSV searched by “Topics” showed each prefecture and included neighboring prefectures (S1 Table). The “Top related queries” attributes of RSV searched by “Topics” in Japan and by prefecture showed content relevant to each tree (S2 Table). Most of the “related topics” attributes of RSV searched by “Topics” in Japan and by prefecture showed the relevant place names (S3 Table). In addition, they included mainly neighboring locations, camera, and other famous cherry trees.

## Discussion

### Validity of seasonality of time-series of RSV

The time-series of RSV showed bell-shaped seasonal variability in each year, with the annual maximum during the flowering period (Figs 3–5 and S1–S3). In Japan, the cherry flowering forecast and days of blossoming in each region are reported every day from March to May on television, on radio, in newspapers, and online. Such reports may motivate Japanese people to search for flowering information on famous sites in each region on Google. If they search independently, the annual maximum RSV for each year may converge around the average date of flowering. However, it showed large time lags among years (Figs 3–5 and S1–S3). On the other hand, many famous cherry flowering sites are used in television dramas, literature, and animations, so people interested in such works may search Google for reasons unrelated to cherry flowering season. These searches may introduce noise in the seasonal variability of RSV. To rule out such searches, the “Top related queries” and “Top related topics” attribute information of RSV must be examined, although we did not find results unrelated to cherry flowering season in our analysis.

The time-series of RSV in all Japan (national scale) showed more clear seasonality than those by prefecture (regional scale) (Figs 3–5 and S1–S3). This difference may be due to differences in population size. Fukushima, Yamanashi, and Gifu each have from 0.8 million to 1.95 million inhabitants [36–38], whereas Japan has 125.05 million (as of 1 May 2022; [39]). This explanation is supported by our finding that the “Interest by region” attribute of RSV searched in all Japan included many neighboring prefectures (S1 Table). Interestingly, the frequency of appearance of large cities such as Tokyo, Kanagawa, Aichi, and Osaka was not high in the “Interest by region” attribute information (S1 Table), maybe because the large cities have many famous cherry flowering sites in local neighborhoods (e.g., avenues and parks), so city residents may have less interest in the three ancient trees than residents in each prefecture. However, it remains uncertain whether the seasonality of RSV represents regional interest in cherry flowering or overall Internet media coverage.

The seasonality of RSV and the correspondence between the first date of annual maximum RSV and the first date of full bloom based on the flowering information published on websites became clearer after changes in the GT algorithms (Figs 4–6 and S2 and S3). This finding suggests that the time-series of RSV include uncertainty due to the data collection design in GT. The accuracy of the time-series of RSV from 2004 to 2015 as a proxy for year-to-year variability of cherry flowering may be lower than that from 2016 to 2021.

### Applicability of GT to other cherry flowering sites

To validate the usability of GT for cherry trees in other regions, we examined the time-series of RSV at 41 sites where cherry trees are designated as national scenic sites or natural treasures [26]. Eleven sites showed clear seasonality of RSV searched by “Topics.” However, many other famous sites are not so designated and yet people still searched for them. Therefore, designation as a national treasure is not important, but fame is. These findings suggest that RSV of searches for famous cherry flowering sites in regions may be a proxy for flowering phenology. In fact, we found clear seasonality of the time-series of RSV searched by “Topics” in relation to famous sites such as Hirosaki Park in Aomori (40°36′27.8″N, 140°27′51.7″E); Hitome Senbonzakura along the Shiroishi River in Miyagi (38°03′31.3″N, 140°45′46.5″E); the Gongendo River in Saitama (36°05′31.1″N, 139°43′24.0″E); Ueno Onshi Park (35°42′55.7″N, 139°46′26.6″E) and Chidorigafuchi in Tokyo (35°41′24.5″N, 139°44′54.8″E); Shokawazakura Park in Gifu (36°05′25.3″N, 136°56′20.9″E), Cherry Avenue along the Asuwa River in Fukui (36°03′49.1″N, 136°12′30.6″E); Hirano Shrine (35°01′57.2″N, 135°43′55.1″E) and Maruyama Park in Kyoto (35°00′12.8″N, 135°46′49.9″E); and Mount Yoshino in Nara (34°21′23.0″N 135°52′14.1″E). We also found it in the Tidal Basin in Washington, DC, USA (38°53′02.6″N, 77°02′18.5″W), which is famous for the National Cherry Blossom Festival [40].

## Conclusions

The seasonality of time-series of RSV obtained from GT represented the flowering phenology of three famous cherry trees in Japan. The year-to-year variability of the dates of the maximum RSV tended to correspond to that of the first date of full bloom. Our approach may be useful for application to multiple famous cherry flowering sites for which long-term detailed observation data are not available. In addition, it may indirectly provide useful information on spatiotemporal variability of flowering phenology in Japan for international people who cannot easily find information on flowering phenology on websites and micro-blogs written in Japanese. Despite the dependence of people’s interest in culture by region or country, GT offers new possibilities for phenological studies for examining seasonal foods and uses of seasonal plants and animals.

## Supporting information

S1 Fig For Miharu Takizakura, the relationship between time-series of RSV searched by “Topics” in all Japan and Fukushima and flowering information published on websites from 2016 to 2018. mv. ave.: moving average.
(TIFF)

S2 Fig For Yamataka Jindaizakura, the relationship between time-series of RSV searched by “Topics” in all Japan and Yamanashi and flowering information published on websites from 2004 to 2014. mv. ave.: moving average.
(TIFF)

S3 Fig For Neodani Usuzumizakura, the relationship between time-series of RSV searched by “Topics” in all Japan and Gifu and flowering information published on websites from 2004 to 2019. mv. ave.: moving average.
(TIFF)

S1 Table “Interest by region” attribute information of RSV searched by “Topics” in all Japan (when RSV ≥ 30). (DOCX)

S2 Table “Top related queries” attribute information of RSV searched by “Topics” in all Japan and by prefecture (when RSV ≥ 30). Original attribute information is provided in Japanese.
(DOCX)

S3 Table “Top related topics” attribute information of RSV searched by “Topics” in all Japan and by prefecture (when RSV ≥ 30). (DOCX)
