# Supplementary figures and images for: Monitoring of cherry flowering phenology with Google Trends
Source: PLoS One. 2022 Jul 21;17(7):e0271648. doi: 10.1371/journal.pone.0271648 (PMC9302780; doi:10.1371/journal.pone.0271648)

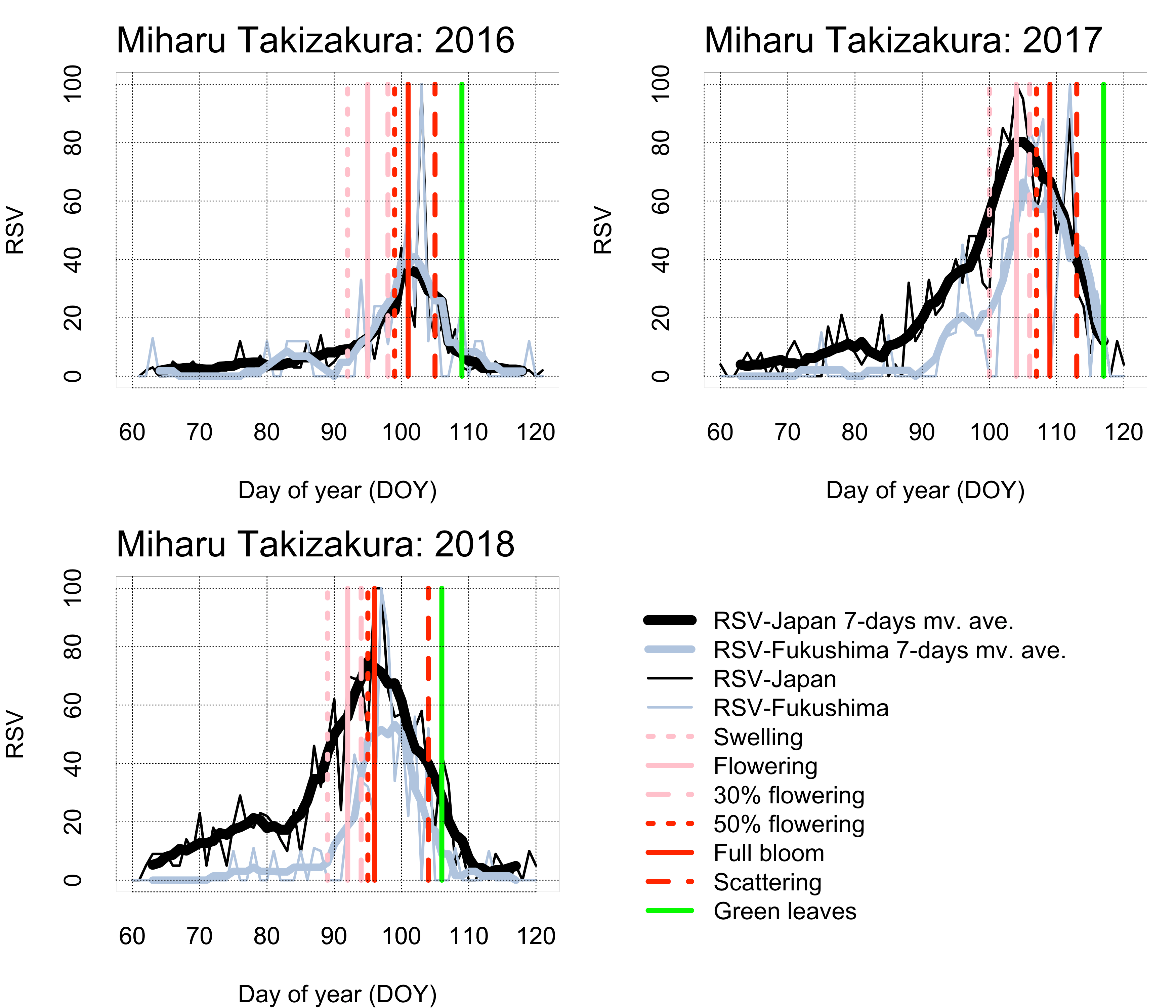

Supplement: S1 Fig — mv. ave.: moving average. (TIFF) [file pone.0271648.s001.tiff]
