# Supplementary material for: Monitoring of cherry flowering phenology with Google Trends
Source: PLoS One. 2022 Jul 21;17(7):e0271648. doi: 10.1371/journal.pone.0271648 (PMC9302780; doi:10.1371/journal.pone.0271648)
Supplement: S1 Table — (DOCX) [file pone.0271648.s004.docx]

**Supporting information**

Table S1 “Interest by region” attribute information of RSV searched by “Topics” in all Japan (when RSV ≥ 30).

| Tree | “Interest by region” attribute information |
| --- | --- |
| Miharu Takizakura, Fukushima | Akita (1), Aomori (1), Hiroshima (1), Ishikawa (1), Iwate (1), Kagoshima (1), Shizuoka (1), Yamanashi (1), Saitama (2), Yamagata (2), Miyagi (4), Niigata (4), Gunma (5), Tochigi (7), Ibaraki (9), Fukushima (16) |
| Yamataka Jindaizakura, Yamanashi | Aichi (1), Chiba (1), Hokkaido (1), Niigata (1), Tochigi (1), Shizuoka (2), Kanagawa (3), Gunma (4), Nagano (4), Yamanashi (11) |
| Neodani Usuzumizakura, Gifu | Akita (1), Fukui (1), Fukuoka (1), Gunma (1), Kagawa (1), Kumamoto (1), Kyoto (1), Oita (1), Shimane (1), Tochigi (1), Tokushima (1), Wakayama (1), Yamaguchi (1), Niigata (2), Shiga (2), Nagano (3), Nara (3), Shizuoka (4), Ishikawa (5), Toyama (5), Aichi (8), Mie (9), Gifu (17) |

Numbers in parentheses show the numbers of years in which the terms appeared.
