# Supplementary material for: Monitoring of cherry flowering phenology with Google Trends
Source: PLoS One. 2022 Jul 21;17(7):e0271648. doi: 10.1371/journal.pone.0271648 (PMC9302780; doi:10.1371/journal.pone.0271648)
Supplement: S2 Table — Original attribute information is provided in Japanese. (DOCX) [file pone.0271648.s005.docx]

**Supporting information**

Table S2 “Top related queries” attribute information of RSV searched by “Topics” in all Japan and by prefecture (when RSV ≥ 30). Original attribute information is provided in Japanese.

| Site | Target area | “Top related queries” attribute information |
| --- | --- | --- |
| Miharu Takizakura, Fukushima | Japan | 三春/Miharu, 滝の/taki-no, 滝/taki, 桜/zakura, ライブ/live, カメラ/camera |
|  | Fukushima | 三/Mi, 春の/haruno, 三春/Miharu, 滝/taki, 桜/zakura, ライブ/live, カメラ/camera |
| Yamataka Jindaizakura, Yamanashi | Japan | 神代/Jindai, 桜/zakura, 山高/Yamataka, 山梨/Yamanashi |
|  | Yamanashi | 神代/Jindai, 桜/zakura |
| Neodani Usuzumizakura, Gifu | Japan | 薄墨/Usuzumi, 淡/Usu, 墨/zumi, 桜/zakura |
|  | Gifu | 薄墨/Usuzumi, 桜/zakura |
