# Supplementary material for: Monitoring of cherry flowering phenology with Google Trends
Source: PLoS One. 2022 Jul 21;17(7):e0271648. doi: 10.1371/journal.pone.0271648 (PMC9302780; doi:10.1371/journal.pone.0271648)
Supplement: S3 Table — (DOCX) [file pone.0271648.s006.docx]

**Supporting information**

Table S3 “Top related topics” attribute information of RSV searched by “Topics” in all Japan and by prefecture (when RSV ≥ 30).

| Site | Target area | “Top related topics” attribute information |
| --- | --- | --- |
| Miharu Takizakura, Fukushima | Japan | Miharu, Fukushima |
|  | Fukushima | Miharu |
| Yamataka Jindaizakura, Yamanashi | Japan | Yamanashi, Kuonji (neighboring famous temple), Anthesis, Hokuto (location of Yamataka Jindaizakura) |
|  | Yamanashi | – |
| Neodani Usuzumizakura, Gifu | Japan | Gifu, Motosu (location of Neodani Usuzumizakura), Neo (location of Neodani Usuzumizakura), Miharu Takizakura, Jindai-zakura, Anthesis, Takehara (place name in Japan), Camera |
|  | Gifu | – |

Meaning of words is shown in parentheses.
